# Self-Medication Practice Among Older Adults: Prevalence and Associated Factors

**DOI:** 10.1093/geroni/igaa057.676

**Published:** 2020-12-16

**Authors:** Medea Locquet, Charlotte Beaudart, Jean-Yves Reginster, Olivier Bruyère

**Affiliations:** 1 University of Liège, Liège, Belgium; 2 University of Liège, liege, Belgium

## Abstract

The self-medication practice, or the self-administration of medicine or dietary supplements without any physician’s advice, represents a public health issue due to its potentially related adverse health events. In older adults, few data are available on self-medication behaviors. The main objective of our study was thus to assess the prevalence of self-medication in a population of older adults. As a secondary exploratory analysis, we seek to determine the determinants of self-medication practice and its association with adverse health outcomes. Our sample was comprised of men and women aged 65 and over from the SarcoPhAge study. Data regarding self-administration of substances were collected through self-administered questionnaire. Clinical and socio-demographic characteristics of the participants as well as medical history were recorded by the investigator. A total of 272 older adults were recruited with a mean age of 77.3±5.2 years. Among them, 119 (43.8%) reported resorting to self-medication (38.6% self-administration of vitamins and dietary supplements, 28.5% self-administration of drugs). Participants which resort to self-medication were older (78.9±4.7 years versus 77.1±3.9 years, p=0.04) and had a lower body mass index (25.7±3.9 versus 27.3±5.2, p=0.006). After adjustments for confounding factors, significant associations were noted between self-medication practice and fractures (OR=3.36[1.02-11.28]) and hospitalizations (OR=1.77 [1.03-3.05]) occurred during the previous year. The practice of self-medication seems widespread among the older age. It seems more frequent in more vulnerable subjects and having experienced adverse health outcomes. This type of health practice must therefore attract the attention of health actors.

